# Salvianic acid A alleviates chronic alcoholic liver disease by inhibiting HMGB1 translocation via down‐regulating BRD4

**DOI:** 10.1111/jcmm.15473

**Published:** 2020-06-29

**Authors:** Yanwen Lan, Ran Yan, Wen Shan, Junyi Chu, Ruimin Sun, Ruiwen Wang, Yan Zhao, Zhanyu Wang, Ning Zhang, Jihong Yao

**Affiliations:** ^1^ Department of Pharmacy The Second Hospital of Dalian Medical University Dalian China; ^2^ Department of Pharmacology Dalian Medical University Dalian China; ^3^ Department of Pharmacy Dalian Seventh People's Hospital Dalian China; ^4^ Department of Pharmacy The Third Hospital of Dalian Medical University Dalian China; ^5^ Department of General Surgery The Second Affiliated Hospital of Dalian Medical University Dalian China

**Keywords:** alcoholic liver disease, BRD4, HMGB1, inflammation, SAA

## Abstract

Alcoholic liver disease (ALD) is the major cause of chronic liver disease and a global health concern. ALD pathogenesis is initiated with liver steatosis, and ALD can progress to steatohepatitis, fibrosis, cirrhosis and even hepatocellular carcinoma. Salvianic acid A (SAA) is a phenolic acid component of Danshen, a Chinese herbal medicine with possible hepatoprotective properties. The purpose of this study was to investigate the effect of SAA on chronic alcoholic liver injury and its molecular mechanism. We found that SAA significantly inhibited alcohol‐induced liver injury and ameliorated ethanol‐induced hepatic inflammation. These protective effects of SAA were likely carried out through its suppression of the BRD4/HMGB1 signalling pathway, because SAA treatment largely diminished alcohol‐induced BRD4 expression and HMGB1 nuclear translocation and release. Importantly, BRD4 knockdown prevented ethanol‐induced HMGB1 release and inflammatory cytokine production in AML‐12 cells. Similarly, alcohol‐induced pro‐inflammatory cytokines were blocked by HMGB1 siRNA. Collectively, our results reveal that activation of the BRD4/HMGB1 pathway is involved in ALD pathogenesis. Therefore, manipulation of the BRD4/HMGB1 pathway through strategies such as SAA treatment holds great therapeutic potential for chronic alcoholic liver disease therapy.

## 1 INTRODUCTION

The liver is an imperative organ responsible for metabolic function and plays a unique role in the human body. However, the liver is more susceptible to a poor lifestyle, habits such as drug and alcohol abuse, and environmental factors such as contaminants than many other organs in humans.^1^ Alcoholic liver disease (ALD) is the leading cause of chronic liver disease and a serious health problem worldwide. ALD pathogenesis progresses through a wide spectrum of diseases including alcoholic steatosis, hepatitis, fibrosis and cirrhosis.^2^ Alcohol can increase metabolic pressure and causes oxidative stress, lipid peroxidation and inflammation damage to the liver in 10%‐40% of heavy drinkers.^3^ Many studies, including ours, have confirmed that inflammation is a pivotal event in several types of liver injury, including ALD.^4^, ^5^ Therefore, an in‐depth understanding of the mechanism of alcoholic liver inflammation may be clinically significant for the prevention and treatment of alcoholic liver disease.

BRD4, a member of the bromo and extraterminal (BET) family of genes, functions as a transcriptional coactivator through its bromodomain to carry out various pathophysiological activities.^6^, ^7^ Accumulating evidence indicates that BRD4 is a positive regulator that promotes inflammatory responses. For example, BRD4 inhibition attenuated the inflammatory response in microglia and facilitated recovery after spinal cord injury in rats.^8^ In addition, BRD4 suppression alleviated cerebral ischaemia‐induced brain injury via inhibiting inflammatory activities.^9^ Mechanistically, BRD4 regulates expression of the inflammatory gene enhancer RNA (eRNA) and participates in its synthesis.^10^, ^11^ However, whether BRD4 is involved in regulating alcohol‐induced inflammation during ALD is unclear.

High‐mobility group box protein 1 (HMGB1), a highly conserved multifunctional nuclear protein, is related to regulating gene transcription and maintaining nucleosome structure.^12^ Recent studies have shown that HMGB1 is a highly conserved multifunctional nuclear protein; however, nuclear HMGB1 is released into the extracellular space during inflammatory responses.^13^, ^14^, ^15^ Acting as an inflammatory cytokine itself, HMGB1 suppression and translocation blockade were reported to protect against non‐alcoholic steatohepatitis in our previous study.^16^ More importantly, HMGB1 was recently reported to be an immediate object of BRD4 in osteoarthritis.^17^ Therefore, we suggest that the BRD4/HMGB1 pathway is involved in the regulation of ALD pathogenesis and that the BRD4/HMGB1 pathway represents a potential target for anti‐inflammatory therapy in ALD.

**Figure 1:**
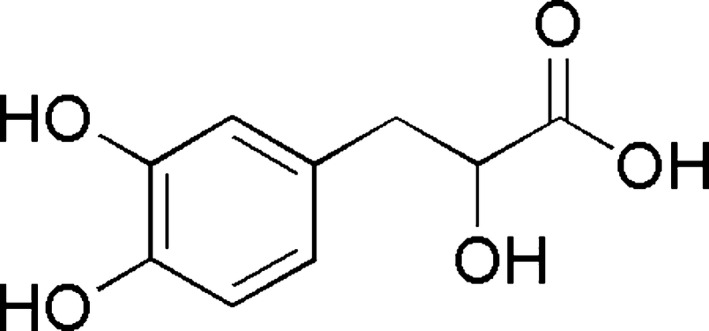
Chemical structure of salvianic acid A

Besides abstinence from alcohol and liver transplantation, there are no efficient therapies to prevent the pathogenesis of ALD.^18^ In the absence of a reliable liver‐protective drug in modern medicine, many herbal extracts and natural products have been found to be possible drugs to treat various chronic liver diseases with relatively high efficiency and low toxicity.^19^ Danshen, a water‐soluble bioactive distilled extract derived from the dried root of *Salvia miltiorrhiza* Radix, has been widely used in many Asian countries over thousands of years for the treatment of heart diseases and cerebrovascular diseases.^20^, ^21^ Salvianic acid A (SAA; Figure 1) is an abundant and structurally representative water‐soluble active component of Danshen.^22^ Recent research has suggested that SAA exhibits liver‐protective effects in the treatment ALD^23^, ^24^; however, the underlying molecular mechanisms of these effects have not been reported.

In the recent research, we validated the protective effects of SAA on chronic alcoholic liver disease using a well‐established rat ALD model and discovered that SAA exerts its liver‐protective effects through, at least partially, suppressing alcohol‐induced activation of the BRD4/HMGB1 inflammatory pathway in the rat liver.

## 2 MATERIALS AND METHODS

### 2.1 Chemicals

SAA (purity > 98%) was purchased from Guizhou Jingfeng Injection Co., Ltd. (Guizhou, China). MEM and foetal bovine serum were purchased from Life Technologies (Carlsbad, CA, USA). All biochemical indicator kits and other chemicals were commercially available.

### 2.2 Animals and treatments

Male Wistar rats weighing 180 to 220 g (6 weeks old) were obtained from the Experimental Animal Center of Dalian Medical University (SCXK 2008‐0002). All animal maintenance and treatment procedures were in concordance with the Guide for the Care and Use of Laboratory Animals from the National Institutes of Health and had been authorized by the Institutional Animal Committee of Dalian Medical University. All animals with standard chow and water ad libitum were housed under standard laboratory conditions for approximately one week. The rats were nourished this way: (1) control, (2) control + SAA (40 mg/kg/d), (3) ethanol, (4) ethanol + SAA (20 mg/kg/d) and (5) ethanol + SAA (40 mg/kg/d). Rats in the SAA group received SAA (20 and 40 mg/kg/d) by intragastric administration every day, and the same volume of normal saline was administered to rats in the control group. After exposure to the Lieber‐DeCarli ethanol diet^25^ for 8 weeks, all the rats were killed at the end of the experiment. Blood samples were obtained from the abdominal aorta, and liver tissues were gathered and snap‐frozen on liquid nitrogen before being stored at −80°C until use.

### 2.3 Biochemical assays

Serum was separated from the blood samples by centrifugation at 3000 rpm for 15 minutes. The serum levels of triglyceride (TG), total cholesterol (TC), alanine aminotransferase (ALT) and aspartate aminotransferase (AST) were determined using commercial kits (Nanjing Jiancheng Bioengineering Institute, China) following the manufacturer's instructions.

### 2.4 Liver histomorphology

ALD liver samples and normal controls were collected from the Second Hospital of Dalian Medical University. All procedures that involved human samples were approved by the Second Hospital of Dalian Medical University Review Board (Dalian, China) and were consistent with the principles outlined in the Declaration of Helsinki.

Liver tissues were stained with haematoxylin and eosin (H&E) and Oil Red O staining that was used to recognize tissue lipidosis. Nile red solution (1 μg/mL), a selective fluorescent stain, was used to determine intracellular lipid droplets. Lipid‐bound Nile red was assayed with a fluorescence microscope.

### 2.5 Cell culture and treatment

The AML‐12 mouse hepatocyte cell line was purchased from American Type Culture Collection (Rockefeller, USA). The cells were treated with 10 μmol/L SAA for 6 hours, followed by exposure to 100 mmol/L ethanol for 24 hours.

### 2.6 Immunofluorescence staining

After fixed in 4% formaldehyde, the 1% bovine serum albumin in 0.1% Triton X‐100 was used to block cells that were hatched with primary antibodies at 4°C overnight. The cells were hatched with the appropriate Cy3‐ or FITC‐conjugated secondary antibodies for 2 hours at room temperature and then counterstained with DAPI (Beyotime Institute of Biotechnology, Hangzhou, China). The following antibodies were used: anti‐BRD4 monoclonal antibody, anti‐HMGB1 monoclonal antibody, FITC‐conjugated AffiniPure goat anti‐rabbit IgG (H + L) and Cy3‐conjugated AffiniPure goat anti‐rabbit IgG (H + L). All the antibodies were purchased from Proteintech (Wuhan, China). Colorimetric analysis was carried out by Vischeck software.

### 2.7 Preparation of nuclear and cytosolic fractions

Nuclear and cytosol liver extracts were prepared with a Nuclear and Cytoplasmic Protein Extraction Kit (Beyotime Institute of Biotechnology, Shanghai, China) in accordance with the manufacturer's protocols. All steps were carried out on ice or at 4°C unless stated otherwise.

### 2.8 Western blot analysis

Equal amounts of protein were resolved by 8%‐12% SDS‐PAGE and transferred to PVDF membranes. After blocking with 5% non‐fat dry milk in Tris‐buffered saline, the membranes were incubated overnight with primary antibodies against BRD4, HMGB1, IL‐1β, TLR4 and β‐actin. Specific bands were detected by enhanced chemiluminescence (ECL) method using a Bio Spectrum gel imaging system (UVP, USA).

### 2.9 RNA isolation and real‐time PCR

After treatment, total RNA was extracted from liver samples and AML‐12 cells using TRIzol reagent (Invitrogen, CA, USA) according to the manufacturer's instructions. First‐strand cDNA was generated using 1 µg of total RNA as a template with the PrimeScriptTM RT Reagent Kit (TaKaRa, Japan). RNA was amplified with SYBR Premix Ex TaqTM II (TaKaRa). Expression levels in each sample were determined by calculating ΔΔCt, which standardized to β‐actin levels. The primer sequences are shown in Table 1.

**Table 1:** Primer sequences

Gene	Forward primer	Reverse primer
BRD4 rat	GTGGGAGGAAAGAAACAGGGACA	AGGAGGAGGATTCGGCTGAGG
TNF‐α rat	CAAGAGCCCTTGCCCTAAGG	CGGACTCCGTGATGTCTAAGTACTT
TNF‐α mouse	ACAAGGCTGCCCCGACTAC	TGGGCTCATACCAGGGTTTG
IL‐6 rat	CTGATTGTATGAACAGCGATGATG	GGTAGAAACGGAACTCCAGAAGAC
IL‐6 mouse	ACCACTCCCAACAGACCTGTCT	CAGATTGTTTTCTGCAAGTGCAT
IL‐1β rat	CCCAAGCACCTTCTTTTCCTT	TCAGACAGCACGAGGCATTT
IL‐1β mouse	CTTTCCCGTGGACCTTCCA	CTCGGAGCCTGTAGTGCAGTT
β‐actin rat	GGAAATCGTGCGTGACATTAAAG	CGGCAGTGGCCATCTCTT
β‐actin mouse	AGAGGGAAATCGTGCGTGAC	CAATAGTGATGACCTGGCCGT

### 2.10 RNA silencing experiment

AML‐12 cells were seeded onto 6‐well plates at a density of 1 × 105 cells per well. When the cells had reached 50%‐60% confluence, they were transfected with 100 nmol/L BRD4 siRNA, HMGB1 siRNA or non‐binding control siRNA using Lipofectamine 3000 (Invitrogen, Karlsruhe, Germany) according to the manufacturer's instructions. The following siRNA sequences were used: BRD4 siRNA: sense 5′‐GCC UGA GAU GAA GCC UGU ATT‐3′, antisense 5′‐UACAGGCUUCAUCUCAGGCTT‐3'; HMGB1 siRNA: sense 5′‐GGAUAUUGCUGCCUACAGATT‐3', antisense 5′‐UCUGUAGGCAGCAAUAUCCTT‐3; and negative control siRNA: sense 5′‐UUCUCCGAACGUGUCACGUTT‐3′, antisense 5′‐ACGUGACACGUUCGGAGAATT‐3′ (GenePharma, Shanghai, China).

### 2.11 Statistical analysis

The data are expressed as the mean ± standard deviation (SD). Data were analysed with GraphPad Prism 5 software (GraphPad Software, Inc, San Diego, CA). The unpaired Student's *t* test or non‐parametric Mann‐Whitney *U* test was used for statistical analysis between two groups. For comparisons of multiple groups, one‐way ANOVA and the non‐parametric Kruskal‐Wallis test were performed, followed by Dunnett's multiple comparison post hoc test. Differences were considered statistically significant when *P* < .05.

## 3 RESULTS

### 3.1 SAA attenuates chronic alcohol‐induced liver injury

**Figure 2:**
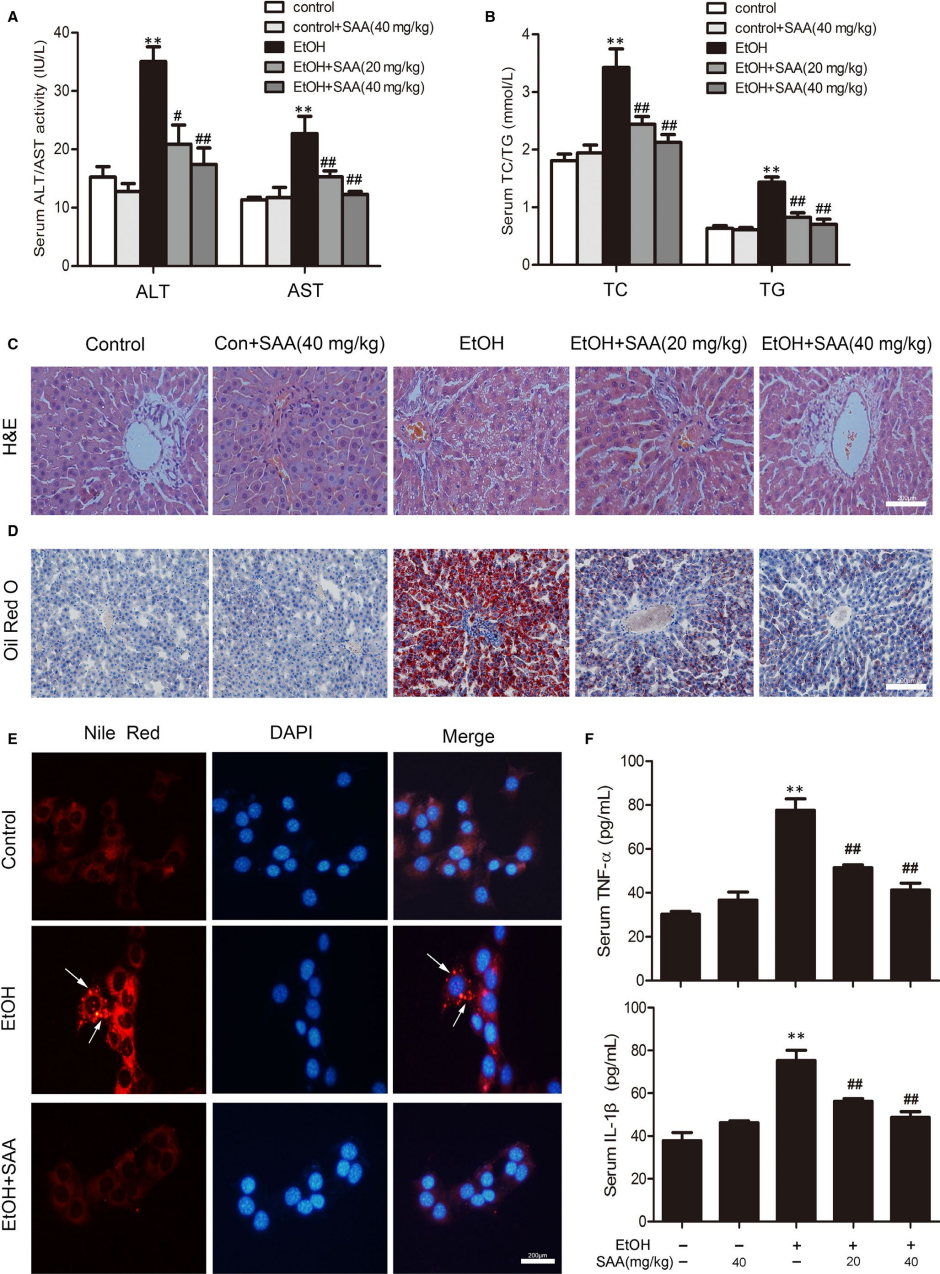
SAA diminishes alcohol‐induced liver injury and hepatic steatosis. A, Serum levels of ALT and AST (n = 8). B, Serum levels of TC and TG. The results are the mean ± SD (n = 8). C, D, H&E staining and Oil Red O staining of liver sections from the experimental groups: a, control; b, control + SAA (40 mg/kg); c, ethanol; d, ethanol + SAA (20 mg/kg); and e, ethanol + SAA (40 mg/kg). H&E and Oil Red O staining. Scale bar, 200 μm. E, Nile red staining: control group; ethanol group (100 mmol/L ethanol for 24 h); ethanol group pre‐treated with 10 µmol/L SAA. Scale bar, 200 μm. F, Serum TNF‐α and IL‐1β. The results are the mean ± SD (n = 8), **P < .01 vs the control group; ^#^ *P* < .05, ^##^ *P* < .01 vs the ethanol group

We first investigated whether SAA treatment protects against chronic alcohol‐induced liver injury in rats. As expected, the serum levels of ALT and AST were significantly increased in rats 8 weeks after an ethanol diet. SAA observably suppressed ALT and AST activities in a dose‐dependent manner compared with those in the ethanol group (Figure 2A), indicating that SAA reveals a hepatoprotective effect against ALD. Similarly, while serum TC and TG levels were markedly up‐regulated compared with the control group without alcohol feeding, SAA largely diminished this increase in TC and TG levels in a dose‐dependent manner (Figure 2B). Further histological evaluation by both H&E staining and Oil Red O staining of liver sections displayed that the control treatment produced no apparent abnormalities. Notably, SAA treatment significantly protected rats from ethanol‐induced liver pathogenesis (Figure 2C and D). We then further validated the liver‐protective effects of SAA using AML‐12 mouse hepatocytes. The accumulation of lipid droplets in ethanol‐treated AML‐12 cells was detected by Oil Red O staining, and the number of these droplets was decreased by SAA treatment (Figure 2E). Levels of the inflammatory mediator TNF‐α and IL‐1β are often elevated in liver tissues from individuals with chronic alcoholism.^26^ Similarly, ethanol treatment induced a twofold to threefold increase in both IL‐1β and TNF‐α production. Importantly, to further support our belief that SAA protected rats from alcohol‐induced liver damage, the addition of SAA significantly inhibited ethanol‐induced inflammatory cytokine production (Figure 2F). Hence, these consequences manifest that SAA is effective in protecting the liver from chronic ethanol‐related injury, steatosis and inflammation.

### 3.2 SAA suppresses hepatic inflammation induced by ethanol in vivo and in vitro through inhibiting BRD4 expression

**Figure 3:**
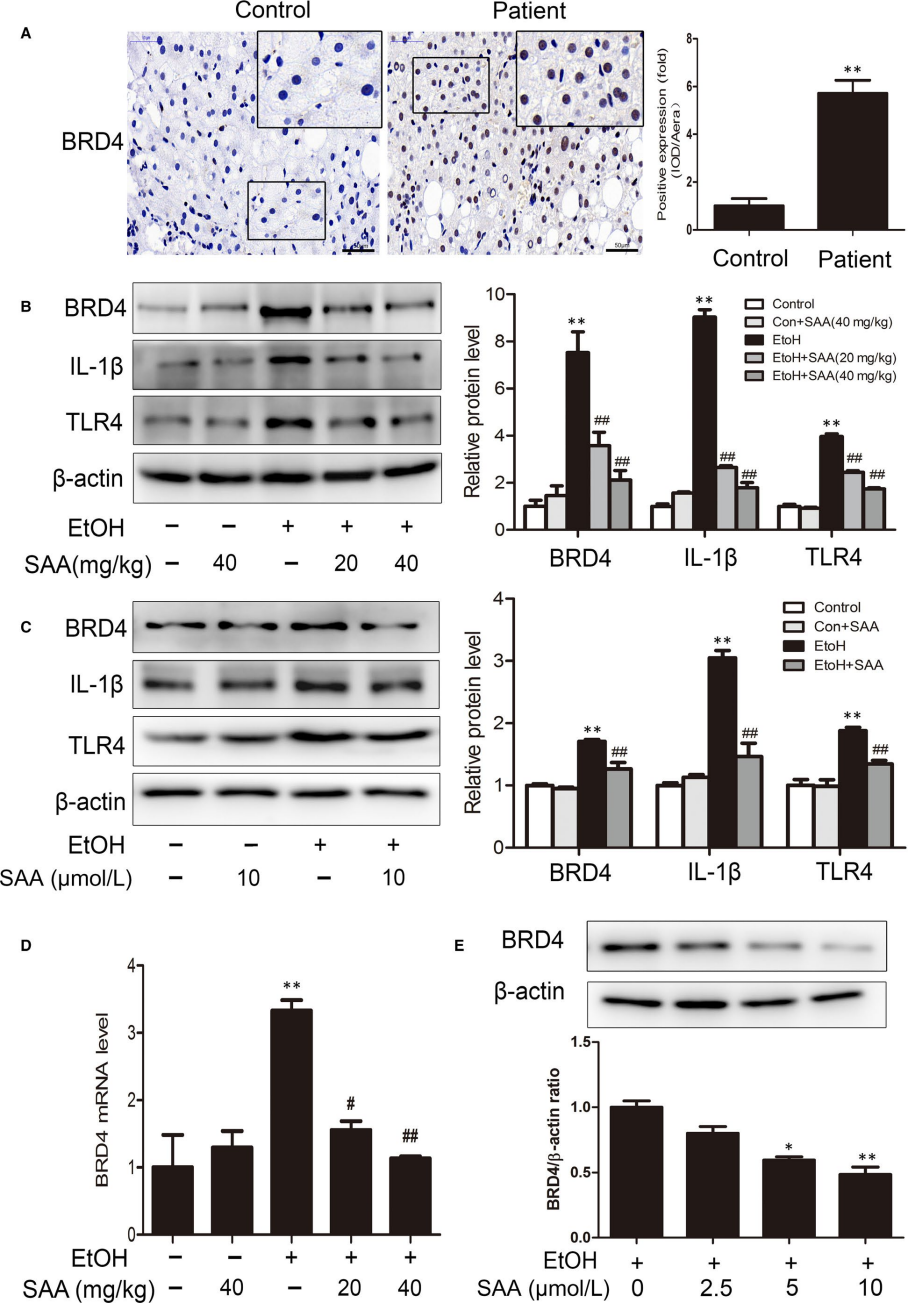
Effects of SAA on the BRD4 protein level in vivo and in vitro. A, IHC staining for BRD4. Scale bar, 50 μm. (n = 3). B, Western blotting analysis of the hepatic BRD4, TLR4 and IL‐1β protein levels in rats (n = 3). C, AML‐12 cells were pre‐treated with SAA (10 μmol/L) for 6 h before exposure to ethanol (100 mmol/L) for 24 h. BRD4, TLR4 and IL‐1β protein expression (n = 3). D, The mRNA levels of BRD4 in rats were measured by real‐time PCR (n = 3). E, AML‐12 cells were pre‐treated with 2.5, 5 or 10 µmol/L SAA for 6 h before exposure to ethanol (100 mmol/L) for 24 h. The figure shows independent response of BRD4 to SAA treatment (n = 3). ^*^ *P* <.05, ^**^ *P* < .01 vs the control group; ^##^ *P* < .01 vs the alcoholic group

BRD4 has latterly been found to be a vital inflammatory mediator, and its activation facilitates the pathogenesis of various diseases.^27^, ^28^ To estimate the remedial potential of targeting BRD4 in ALD, we first assessed the expression level of BRD4 in ALD. As shown in Figure 3A, BRD4 protein levels were significantly up‐regulated in liver tissues from ALD patients compared with normal liver tissues (*P* < .01). We also analysed BRD4 expression in rats and AML‐12 cells upon ethanol treatment. As shown in Figure 3B and C, the expression levels of BRD4 in the rat liver and in AML‐12 cells were significantly increased by ethanol. Notably, SAA treatment largely abolished the increase in BRD4 in the liver tissues of alcohol‐fed rats and AML‐12 cells treated with alcohol. Inflammatory factors such as IL‐1β and Toll‐like receptor‐4 (TLR4) have been suggested to play vital roles in the progression of ALD,^29^, ^30^ and these factors were found to be involved in BRD4‐mediated inflammation.^31^ Indeed, consistent with our observation of altered BRD4 levels, the expression of IL‐1β and TLR4 was significantly increased by ethanol, and SAA treatment largely abolished this increase in IL‐1β and TLR4 in liver tissues from alcohol‐fed rats and AML‐12 cells treated with alcohol (Figure 3B and C). Furthermore, SAA treatment neither affected BRD4 expression in the livers of rats without ethanol feeding used as a control nor altered their inflammatory responses (Figure 3B and C), suggesting that SAA specifically inhibits alcohol‐induced BRD4 expression and its associated inflammation. In addition, we analysed the effect of alcohol BRD4 mRNA level. As shown in Figure 3D, the mRNA level of BRD4 was significantly increased by ethanol, which is largely inhibited by SAA treatment. Furthermore, SAA treatment significantly blunted the alcohol‐induced increase in BRD4 expression in a dose‐dependent manner in AML‐12 cells (Figure 3E). Our results suggested that SAA suppresses hepatic inflammation in chronic alcoholic disease through down‐regulating BRD4 expression.

### 3.3 The BRD4/HMGB1 pathway is related to the regulation of inflammation in ALD

**Figure 4:**
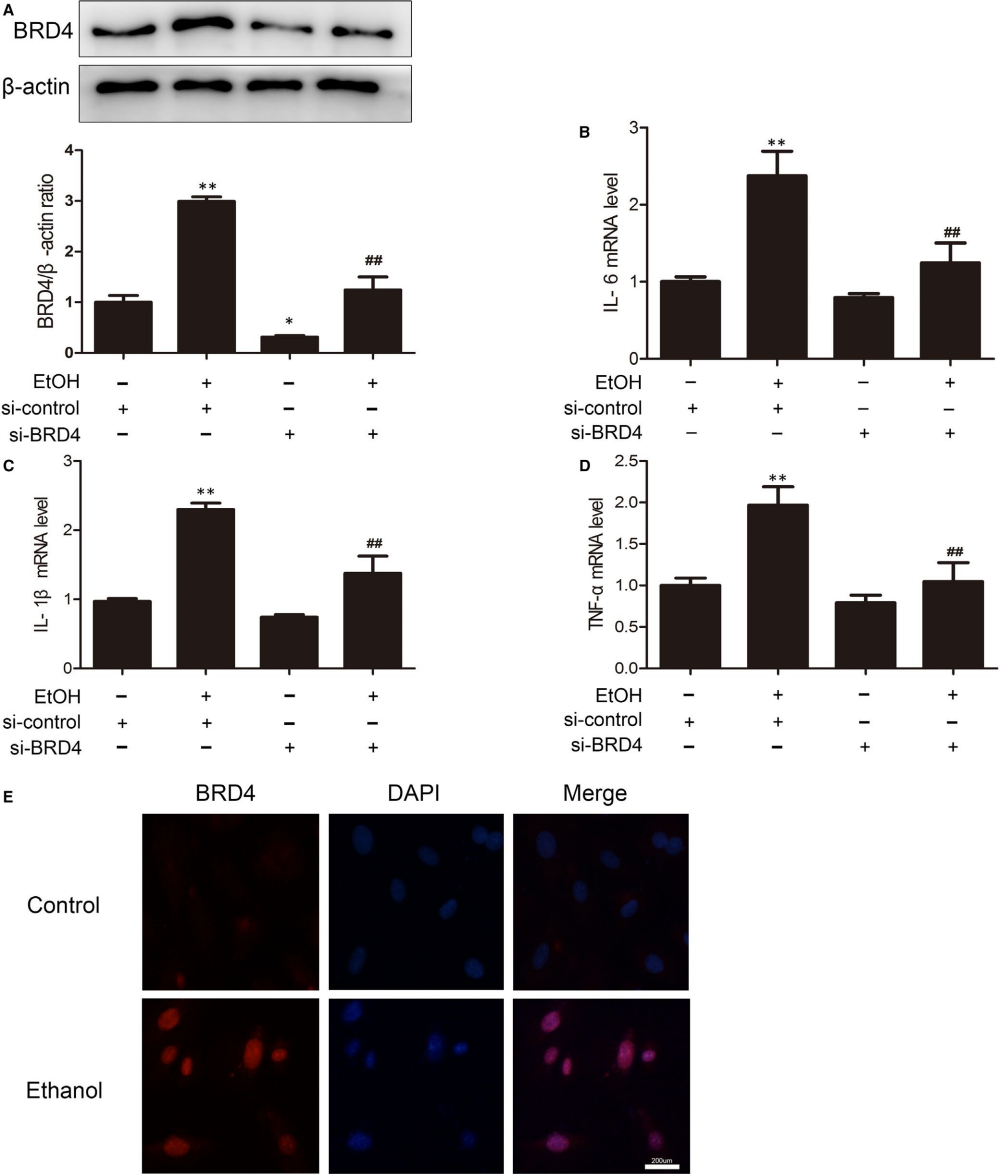
BRD4 knockdown reduces liver inflammation. AML‐12 cells were transfected with control siRNA or BRD4 siRNA for 48 h before exposure to ethanol (100 mmol/L) for 24 h. A, The expression of BRD4 was measured with Western blotting (n = 3). B‐D, The mRNA levels of IL‐6, IL‐1β and TNF‐α in AML‐12 cells were measured by real‐time PCR (n = 3), ^*^ *P* < .05, ^**^ *P* < .01 vs. the si‐control group; ^##^ *P* < .01 vs. the ethanol‐treated group. The levels of (E) immunofluorescence analysis of BRD4 protein expression in AML‐12 cells. Scale bar, 200 μm

Our observation that ethanol‐induced BRD4 expression was inhibited by SAA suggests that SAA protects the liver from alcohol‐mediated liver injury by suppressing BRD4 expression. To further test this speculation, we analysed the impact of the loss of BRD4 function on the ethanol‐induced inflammatory response in vitro. AML‐12 cells treated with ethanol for 24 hours exhibited increased expression of BRD4 (Figure 4A). In addition, immunofluorescence staining validated the increase in BRD4 expression observed via Western blotting (Figure 4E). In accordance with the observed up‐regulation of BRD4 by alcohol, the mRNA levels of the inflammatory factors IL‐1β, IL‐6 and TNF‐α were dramatically enhanced. We then suppressed BRD4 via siRNA to test whether ethanol induces liver inflammation through up‐regulating BRD4. Western blotting validated that BRD4‐specific siRNA, but not control siRNA, inhibited BRD4 expression by more than 80%. Importantly, silencing BRD4 inhibited the ethanol‐stimulated increase in IL‐6, IL‐1β and TNF‐α mRNA levels (Figure 4B, C and D). Together, these results demonstrate that BRD4 plays a crucial role in the progress of ethanol‐induced ALD inflammation.

**Figure 5:**
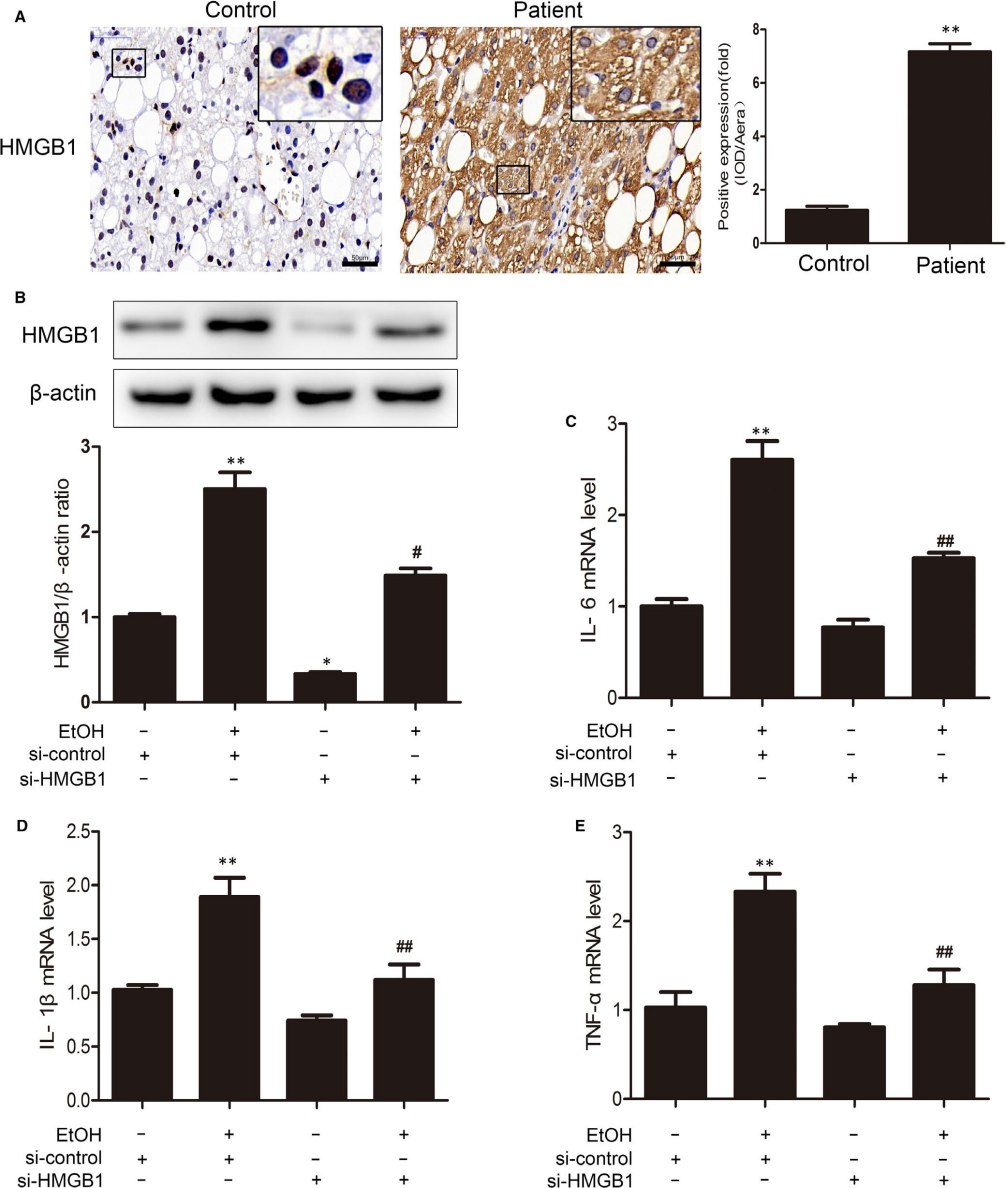
HMGB1 knockdown reduces liver inflammation in vitro. A, IHC staining for HMGB1. Scale bar, 50 μm. (n = 3). AML‐12 cells were transfected with control siRNA or HMGB1 siRNA for 48 h before exposure to ethanol (100 mmol/L) for 24 h. B, The expression of HMGB1 was measured with Western blotting (n = 3). C‐E, The mRNA levels of IL‐6, IL‐1β and TNF‐α in AML‐12 cells were measured by real‐time PCR (n = 3). ^*^ *P* < .05, ^**^ *P* < .01 vs. the si‐control group; ^#^ *P* < .05, ^##^ *P* < .01 vs. the ethanol‐treated group

HMGB1 was recently reported to be an immediate object of BRD4,^17^ suggesting the involvement of HMGB1 in BRD4‐induced inflammation in ALD. To verify the role of HMGB1 in ALD, we first explored the expression levels of HMGB1 in the livers of patients with ALD. As shown in Figure 5A, HMGB1 was detected in the nucleus of normal liver tissue, while the results of ALD patients showed that HMGB1 was most partly transferred from the nucleus to cytoplasm (*P* < .01), implying a pathogenic role of HMGB1 in ALD in humans. In support of this notion, we indeed detected an at least threefold increase in HMGB1 expression induced by ethanol treatment in AML‐12 cells (Figure 5B). Similarly, the mRNA levels of IL‐6, IL‐1β and TNF‐α were up‐regulated threefold to fourfold in AML‐12 cells 24 hours after ethanol treatment (Figure 5B, C, D and E). HMGB1 siRNA was used to knock down HMGB1 in AML‐12 cells to determine whether HMGB1 suppression restores alcohol‐induced inflammation in AML‐12 cells. Notably, the ethanol‐induced enhancement in pro‐inflammatory cytokine expression was largely obstructed by HMGB1 siRNA, indicating that HMGB1 plays a vital role in the development of ethanol‐induced ALD inflammation. Together with the fact that HMGB1 is a downstream target of BRD4,^17^ these results showed that the BRD4/HMGB1 pathway plays an essential role in the regulation of inflammation in ALD.

### 3.4 SAA suppresses HMGB1 nuclear translocation and liberates via the down‐regulation of BRD4 to regulate inflammation in ALD

**Figure 6:**
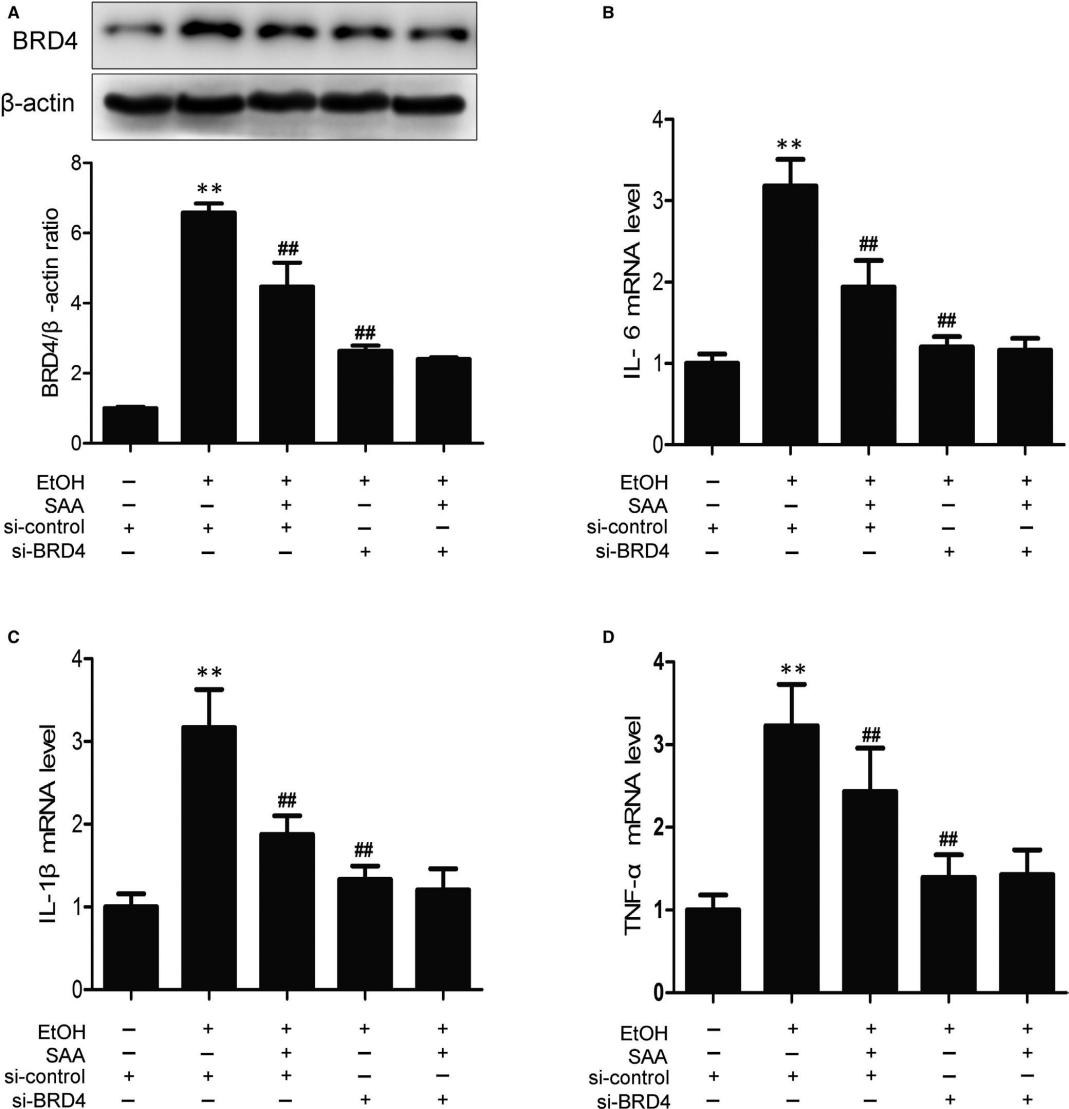
SAA ameliorates the cell injury induced by ethanol through BRD4 pathway. AML‐12 cells were transfected with control siRNA or BRD4 siRNA for 48 h before treatment with SAA (10 μmol/L) for 6 h, and the transfected cells were exposed to ethanol (100 mmol/L) for 24 h. A, The expression of BRD4 was measured with Western blotting (n = 3). B‐D, The mRNA levels of IL‐6, IL‐1β and TNF‐α in AML‐12 cells were measured by real‐time PCR (n = 3). ^**^ *P* < .01 vs. control group, ^##^ *P* < .01, vs. ethanol group

Our data suggested that BRD4 down‐regulation was involved in SAA‐mediated protection against inflammation in rats during ALD pathogenesis. Next, we further investigated the contribution of BRD4 to SAA‐mediated protection in vitro. Similarly, a virtual elevation in BRD4 expression was assessed in AML‐12 cells after 24 hours of ethanol treatment, resulting in a consequent increase in the pro‐inflammatory factors IL‐6, IL‐1β and TNF‐α (Figure 6B, C and D). As expected, SAA treatment significantly down‐regulated BRD4 expression and consequentially diminished alcohol‐induced IL‐6, IL‐1β and TNF‐α expression. siRNA‐mediated BRD4 suppression largely abolished ethanol‐induced inflammatory responses. BRD4 knockdown did not further reduce the inflammatory response in SAA‐treated AML‐12 cells (Figure 6A). Therefore, these results indicate that SAA protection against ethanol‐induced liver inflammation occurs through BRD4 down‐regulation.

**Figure 7:**
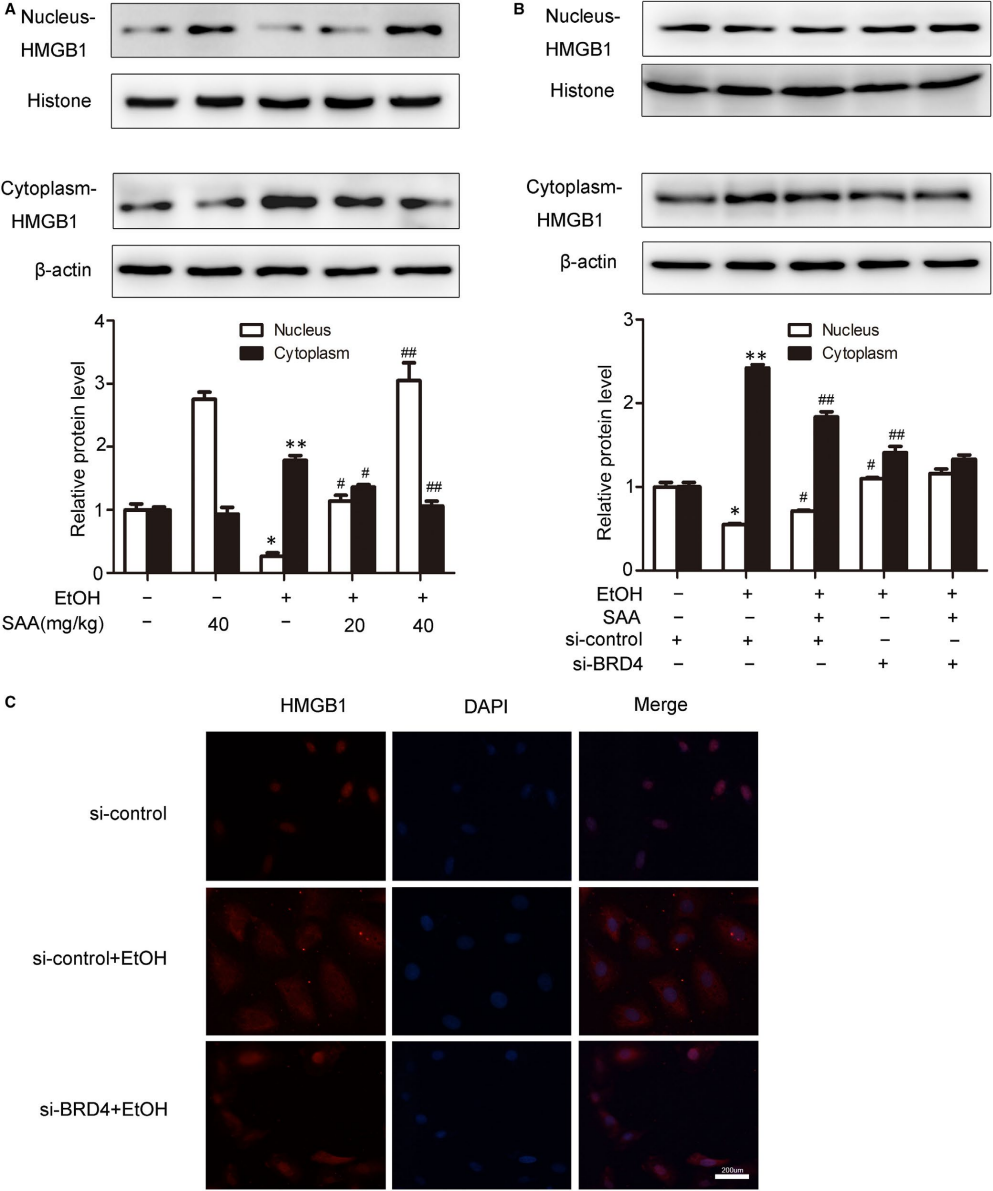
SAA inhibits HMGB1 nuclear translocation and release through regulation of BRD4. A, The levels of nuclear and cytoplasmic HMGB1 in rats livers were measured by Western blotting (n = 3). ^*^ *P < *.05, ^**^ *P < *.01 vs. the control group, ^#^ *P* *< *.05, ^##^ *P* *< *.01 vs. the ethanol group. B, AML‐12 cells were transfected with control siRNA or BRD4 siRNA for 48 h before treatment with SAA (10 μmol/L) for 6 h, and the transfected cells were exposed to ethanol (100 mmol/L) for 24 h. The levels of HMGB1 in nucleus and cytoplasm were evaluated by Western blotting (n = 3). ^*^ *P* *< *.05, ^**^ *P* *< *.01 vs. the si‐control group, ^#^ *P* *< *.05, ^##^ *P < *.01 vs. the si‐control + ethanol group. C, Immunofluorescence analysis of HMGB1 protein expression in AML‐12 cells. Scale bar, 200 μm

Next, we evaluated the efficacy of SAA on the dislocation of HMGB1 in alcohol liver injury. The increased translocation of HMGB1 was surveyed in the livers of ethanol‐fed rats compared with rats in the control group, and this increased translocation was reversed by SAA feeding (Figure 7A). SAA did not alter the HMGB1 subcellular relocation in the livers of rats without ethanol feeding used as a control, suggesting that SAA specifically inhibits the alcohol‐induced cytoplasmic exportation of HMGB1. Consistent with these in vivo observations, both the translocation of HMGB1 and the release of HMGB1 into the supernatant of AML‐12 cells were markedly increased after 24 hours of ethanol exposure (Figure 7B). Moreover, the results showed that after BRD4 siRNA, the levels of expression of HMGB1 exhibited no significant difference in control group; however, ethanol‐induced increased translocation of HMBG1 from the nucleus to the cytoplasm was repressed by BRD4 siRNA. SAA observably depressed this translocation of HMGB1 from the nucleus to the cytoplasm. As expected, SAA treatment did not further inhibit HMBG1 in BRD4 knockdown cells (Figure 7B), clearly indicating that SAA inhibits HMGB1 translocation in a BRD4‐dependent manner in response to inflammation in ALD. Moreover, immunofluorescence staining validated the outcome of Western blotting (Figure 7C). Taken together, our findings indicate that SAA protects against inflammation by inhibiting the nuclear translocation of HMGB1 via down‐regulating BRD4 in ALD.

## 4 DISCUSSION

The current study demonstrates that SAA had potent protective effects in the prevention and treatment of ethanol‐induced liver injury in a rat ALD model and uncovered the novel underlying molecular mechanism of these effects, as documented by the following discoveries: (a) SAA attenuated chronic alcohol‐induced liver injury in both rat and mouse liver cells cultured in vitro. (b) Ethanol induces chronic liver injury partially through up‐regulating BRD4 expression, which consequently promotes BRD4 downstream inflammatory gene expression. (c) SAA achieves its hepatoprotective activity through inhibiting the BRD4‐mediated inflammatory response and hepatic steatosis. (d) Finally, HMGB1 is a BRD4 downstream inflammatory mediator in alcohol‐induced liver injury. Therefore, the BRD4/HMGB1 pathway is involved in alcohol‐induced liver injury, and manipulation of this pathway through strategies such as SAA treatment holds great therapeutic potential for the treatment of alcoholic liver diseases.

*Salvia miltiorrhiza*, also known as Danshen, has been widely used for thousands of years in traditional Chinese medicine with little reported toxicity.^19^ SAA, the most abundant and bioactive water‐soluble compound isolated from Danshen*,* possesses numerous pharmacological effects, such as its anti‐cardiovascular disease, anticoagulative, antimicrobial, antitumour and neuroprotective effects.^20^, ^32^, ^33^ The present study is the first to account that SAA protects rats from chronic alcohol‐induced liver disease, because SAA effectively decreased ALT, AST, TC and TG levels in rats fed an ethanol diet. SAA appears to protect against alcohol‐induced liver injury through suppressing inflammatory factors. Therefore, our study demonstrates that SAA is a favourable therapeutic reagent to treat ALD as well as other liver diseases with an inflammatory response.

The BRD4 gene in the bromo and BET domain family is virtual for inflammatory gene expression, and its suppression has great therapeutic potential in inflammatory disease treatment.^8^, ^9^ Our study reveals for the first time that the expression level of BRD4 is dramatically up‐regulated in alcoholic liver disease. This increase in BRD4 expression was likely responsible for the elevated inflammatory responses in the livers of rats fed ethanol, because BRD4 knockdown largely abolished alcohol‐induced changes in inflammatory responses. Importantly, SAA markedly decreased the alcohol‐induced up‐regulation of BRD4 and elevated inflammatory gene expression in a dose‐dependent manner both in vivo and in vitro. To determine how alcohol stimulation induces BRD4 expression in liver cells, we analysed the effect of alcohol on BRD4 mRNA transcription. As shown in Figure 3D, the mRNA level of BRD4 was significantly increased by ethanol, which is largely inhibited by SAA treatment. These results indicate that BRD4 is induced in hepatocytes by alcohol for inflammatory cytokine production, which consequently leads to liver damage to promote ALD development and progression. SAA treatment protects rats from the disease through suppressing alcohol‐induced BRD4 transcription. We are aware, however, as a protein expression is often regulated at both transcription and post‐translational levels, the possibility that either alcohol or SAA, or both, regulates BRD4 protein stability cannot be fully excluded. Further studies are needed to dissect the molecular puzzles behind ALD development through, at least partially, BRD4‐mediated inflammatory response as well as how SAA achieves its therapeutic efficacy.

HMGB1, a direct target of BRD4,^17^ was originally depicted as a chromatin‐associated protein functioning as a key endogenous danger signalling molecule that has a cytokine‐like extracellular effect on immune cells by advancing pro‐inflammatory signalling and the release of cytokines. In liver I/R and non‐alcoholic liver disease, extracellular HMGB1 was conscientious for the inflammation to hepatic injury.^16^, ^34^, ^35^ When excreted from cells, HMGB1 can also serve as a pro‐inflammatory adjustor or alarmin. In addition, recent studies manifested that the level of HMGB1 was added in ALD, contributing to liver damage.^36^ In most cases, the cells that actively secrete HMGB1 appear to be immune cells, such as macrophages, natural killer cells and dendritic cells. However, it is becoming increasingly clear that non‐immune parenchymal cells also participate in active HMGB1 secretion. The ability of mouse hepatocytes to secrete a large amount of HMGB1 under ethanol treatment in vitro has been validated.^37^ Moreover, alcohol intake enhances HMGB1 expression and translocation from the nucleus to the cytoplasm.^1^ Consistently, the enhancement of HMGB1 expression and translocation from the nucleus to the cytoplasm were detected in rat liver and AML‐12 cells treated with ethanol. Accordingly, anti‐HMGB1 treatment could dramatically decrease the production of cytokines upon alcohol‐induced liver damage, resulting in the suppression of hepatic inflammation. Similar to its effect on BRD4, alcohol treatment induced an efficacious augment in HMGB1 expression.

The activation of HMGB1 is purported to regulate the NF‐κB via the modulation of TLR− 2, 4 and 9, which in turn cascade to regulate the expression of inflammatory mediators, such as IL‐6, IL‐1β and TNF‐α.^38^ Given that IL‐6, pro‐IL‐1β and TNF‐α are abundant pro‐inflammatory cytokines in the progression of liver injury, these pro‐inflammatory cytokines were examined in BRD4/HMGB1‐mediated pathway in ALD. Consistently, increased mRNA expression of IL‐6, IL‐1β and TNF‐α was induced by ethanol in vivo and in vitro and HMGB1 siRNA inhibited the elevated mRNA expression of IL‐6, IL‐1β and TNF‐α in alcohol‐treated AML‐12 cells. Therefore, it can be concluded that BRD4/HMGB1‐mediated inflammatory pathway plays a vital role in ALD. In addition, BRD4 expression is positively correlated to HMGB1 cytoplasmic translocation (Figure 7), which is reduced by either BRD4 knockdown or SAA treatment, suggesting that SAA inhibits HMGB1 translocation in a BRD4‐dependent manner in response to inflammation in ALD.

## 5 CONCLUSIONS

In common, these consequences demonstrate that SAA can prohibit the translocation and release of HMGB1 through down‐regulating BRD4 during ALD. Consistent with these observations, we further demonstrated that the alcohol‐induced up‐regulation and translocation of HMGB1 from the nucleus to the cytoplasm in ALD are dependent on the presence of BRD4. Furthermore, siRNA‐mediated BRD4 knockdown significantly inhibited HMGB1 translocation compared with that following control siRNA treatment, suggesting that BRD4 regulates HMGB1 translocation. Therefore, our study identified the BRD4‐HMGB1‐induced inflammatory response as a previously unappreciated pathway responsible for alcohol‐induced chronic injury and demonstrated that suppression of this pathway by SAA protected rats from ALD.

## CONFLICTS OF INTEREST

The authors declare that there are no conflicts of interest.

## AUTHOR CONTRIBUTION

**Yanwen Lan:** Conceptualization (equal); Data curation (lead); Formal analysis (lead); Investigation (equal); Methodology (lead); Resources (lead); Visualization (lead); Writing‐original draft (lead); Writing‐review & editing (supporting). **Ran Yan:** Conceptualization (equal); Data curation (lead); Formal analysis (equal); Investigation (supporting); Methodology (lead); Resources (equal); Supervision (equal); Validation (lead); Visualization (equal); Writing‐original draft (equal); Writing‐review & editing (equal). **Wen Shan:** Data curation (supporting); Formal analysis (supporting); Methodology (supporting); Resources (supporting); Validation (supporting); Writing‐review & editing (supporting). **Junyi Chu:** Conceptualization (equal); Data curation (equal); Formal analysis (supporting); Resources (supporting); Validation (supporting); Writing‐review & editing (supporting). **Ruimin Sun:** Data curation (supporting); Formal analysis (equal); Methodology (supporting); Resources (supporting); Visualization (supporting). **Ruiwen Wang:** Data curation (equal); Formal analysis (supporting); Methodology (supporting); Resources (supporting); Visualization (supporting). **Yan Zhao:** Data curation (equal); Formal analysis (supporting); Methodology (supporting); Resources (supporting); Visualization (supporting). **Zhanyu Wang:** Data curation (equal); Formal analysis (supporting); Methodology (supporting); Resources (supporting); Visualization (supporting). **Ning Zhang:** Data curation (equal); Funding acquisition (lead); Investigation (lead); Project administration (lead); Resources (lead); Supervision (lead); Validation (lead); Writing‐original draft (lead); Writing‐review & editing (lead). **Jihong Yao:** Data curation (equal); Funding acquisition (lead); Investigation (lead); Project administration (lead); Supervision (lead); Validation (equal); Writing‐original draft (equal); Writing‐review & editing (lead).

## Data Availability

Please contact the authors for data requests.
